# Role of Renal Re-transplantation in ESRD Patients

**DOI:** 10.5812/numonthly.11155

**Published:** 2013-03-30

**Authors:** Alireza Ghadian, Mohammad Hossein Nourbala

**Affiliations:** 1 Nephrology and Urology Research Center, Baqiyatallah University of Medical Sciences, Tehran, IR Iran

**Keywords:** Transplant, Kidney Failure, Chronic

Although there is no data to prove the role of renal re-transplantation, but it is presumed to be a favorable option for many patients after graft loss. Re-transplantation often provides a better survival and good health in comparison with dialysis, that is more obvious for younger recipients and whom receives transplantation either preemptively or shortly after the need for renal replacement therapy has arisen (1). Although some authors believe that for older patients, dialysis is a better option (2). Indeed the type of dialysis doesn’t have any influence on the outcome of transplantation (3).

The etiology of their initial graft loss varies in candidates of kidney re-transplantation depends on the time of graft loss. If graft loss occurred within the first year after transplantation, the most common etiologies are acute rejection and graft thrombosis and if this occurred after 1 year of transplant, chronic rejection accounts for nearly two thirds of the graft loss (4).

In recent decades, kidney re-graft survival has been improved and this improvement is more obvious for grafts from cadaveric source (graft half-life has been increased from 2.0 years in 1988 to 7.5 years in 1995) in comparison with living donors (5) and is suggested that this improvement is the result of improved HLA testing and improvements in immunosuppressive agents (1). 5-year survival after re-transplantation in deceased donors and living donors is as the same as primary transplantation in patients with hypertension, diabetes and renovascular diseases but is less favorable in recipients with polycystic kidney disease (PKD) or glomerular disease (6).

Many variables have an effect on re-transplantation outcome, such as recipient comorbidities, outcome of primary transplant, risk of recurrent disease and the wait time on dialysis before transplantation (1). CKD will occur in the majority of transplanted kidneys that is due to many etiologies include chronic rejection, recurrent disease, calcineurin toxicity and underlying diseases such as diabetes, hypertension and atherosclerosis (1).

Early graft loss after previous transplantation is a predictor of the occurrence of the same process after re-transplantation and so the recipient and potential donors should be evaluated very precisely. This early graft rejection can be due to many factors such as acute rejection, FSGS, IgA nephropathy, MPGN, thrombotic microangiopathy or vascular events (7). Polyoma virus (BK virus) is a known cause of graft loss with increasing incidence and so should be assessed before re-transplantation (8).

The proper time for evaluating recipient and donors for re-transplantation is when the GFR falls to less than 20 mL/min (9, 10). Routine evaluations before re-transplantation are screening for colon, lung, breast, heart, prostate and systemic diseases and malignancies as the same as first transplantation.

Presence of antibodies, that may be created previous blood transfusion, pregnancies or previous transplantation, is a great concern before transplantation (11). Antibodies that are due to graft rejection are absorbed by the graft and after performing nephrectomy for graft loss, they will appear in the plasma with higher concentration and after that will gradually decrease and even may disappear from plasma (12) and evaluation of recipient for these antibodies should be performed with high sensitive assays that will result in avoidance of re-grafting from suspicious donors or using desensitization methods (11). If the patient is highly sensitized to HLA of all potential living donors, the possibility of finding a proper deceased donor is very low, so desensitization is necessary, using high dose gamaglobulin (IVIG) (13) or low dose IVIG with plasma-pheresis (14) and immunosuppression induction with polyclonal antibodies plus low dose IVIG (15). These protocols can be used also for ABO incompatibility (16).

In re-transplantation immunosuppressive agents are used to prevent acute graft loss but risk of infection and malignancies increase. It should be kept in mind that re-transplant patients are at higher risk for acute rejection in comparison with first graft patients (16).

Reasons for previous transplant nephrectomy include: symptoms of graft loss, infections, proteinuria, severe hematuria, the need for more space in the pelvic fossa but in about 80% of these patients, there is no need for previous graft nephrectomy before re-transplantation (16).

In theory, there isn’t any limitation for the number of renal re-transplantation in a patient with ESRD but vascular anatomy can restrict it, so evaluation of the vascular anatomy of recipients before re-transplantation is very important (1, 17).
